# Deep learning and radiomics to predict the mitotic index of gastrointestinal stromal tumors based on multiparametric MRI

**DOI:** 10.3389/fonc.2022.948557

**Published:** 2022-11-23

**Authors:** Linsha Yang, Dan Du, Tao Zheng, Lanxiang Liu, Zhanqiu Wang, Juan Du, Huiling Yi, Yujie Cui, Defeng Liu, Yuan Fang

**Affiliations:** ^1^ Medical Imaging Center, The First Hospital of Qinhuangdao, Qinhuangdao, China; ^2^ Medical Imaging Center, Chongqing Yubei District People’s Hospital, Chongqing, China

**Keywords:** deep learning, radiomics, magnetic resonance imaging, convolutional neural network, gastrointestinal stromal tumor

## Abstract

**Introduction:**

Preoperative evaluation of the mitotic index (MI) of gastrointestinal stromal tumors (GISTs) represents the basis of individualized treatment of patients. However, the accuracy of conventional preoperative imaging methods is limited. The aim of this study was to develop a predictive model based on multiparametric MRI for preoperative MI prediction.

**Methods:**

A total of 112 patients who were pathologically diagnosed with GIST were enrolled in this study. The dataset was subdivided into the development (*n* = 81) and test (*n* = 31) sets based on the time of diagnosis. With the use of T2-weighted imaging (T2WI) and apparent diffusion coefficient (ADC) map, a convolutional neural network (CNN)-based classifier was developed for MI prediction, which used a hybrid approach based on 2D tumor images and radiomics features from 3D tumor shape. The trained model was tested on an internal test set. Then, the hybrid model was comprehensively tested and compared with the conventional ResNet, shape radiomics classifier, and age plus diameter classifier.

**Results:**

The hybrid model showed good MI prediction ability at the image level; the area under the receiver operating characteristic curve (AUROC), area under the precision–recall curve (AUPRC), and accuracy in the test set were 0.947 (95% confidence interval [CI]: 0.927–0.968), 0.964 (95% CI: 0.930–0.978), and 90.8 (95% CI: 88.0–93.0), respectively. With the average probabilities from multiple samples per patient, good performance was also achieved at the patient level, with AUROC, AUPRC, and accuracy of 0.930 (95% CI: 0.828–1.000), 0.941 (95% CI: 0.792–1.000), and 93.6% (95% CI: 79.3–98.2) in the test set, respectively.

**Discussion:**

The deep learning-based hybrid model demonstrated the potential to be a good tool for the operative and non-invasive prediction of MI in GIST patients.

## Introduction

Gastrointestinal stromal tumors (GISTs) are the most common mesenchymal tumors of the digestive tract wall in that they are more common in the stomach and small intestine (1). It is widely believed that GIST originates in Cajal cells, which are involved in gastrointestinal motility (2). GIST occurs at a median age of 60 years (10–100 years), with no sex difference in the distribution (3). Before the advent of tyrosine kinase inhibitors, the most common treatment for most GIST cases was radical surgical resection without any residual tumor. However, even after complete tumor resection, the patients still have a high rate of recurrence and metastasis (4). Another approach for the treatment of GIST was presented through the invention and rational application of targeted drugs, such as imatinib, which significantly improved the recurrence-free survival and overall survival of GIST. The prognosis of GIST is closely related to its risk grade (5). Joensuu and colleagues proposed an improved National Institutes of Health (NIH) grading system to grade the risk of a tumor based on its size, location, mitotic index and whether it is ruptured (6). Different risk grades correspond to different prognoses and treatment methods. For very-low-risk patients, regular follow-up may be used without immediate surgery. For low-risk patients, routine resection similar to benign tumors can be performed without targeted therapy and follow-up. Intermediate- or high-risk patients should receive targeted therapy to shrink the tumor before resection; after surgery, targeted therapy and long-term follow-up should be continued for a period of time (7). Therefore, accurate preoperative assessment of the tumor risk grade has important guiding significance for the treatment plan.

The mitotic index (MI) is an important indicator of GIST risk grading. However, it may be more difficult to perform a preoperative assessment of MI than to obtain morphological information, such as tumor location and size. Pathological examination is still the gold standard to accurately quantify the GIST mitotic index (8). However, as an invasive examination, it may lead to tumor hemorrhage and intraperitoneal spread; hence, a preoperative pathological biopsy is not a routine examination for GIST (9). The application of endoscopic ultrasonography has greatly improved the success rate of preoperative pathological biopsy for mesenchymal tumors. However, a biopsy cannot be performed in some tumors at specific sites (10). For intermediate- or high-risk tumors with active mitosis, preoperative application of the GIST therapy can significantly reduce the tumor size, thus effectively improving the resection rate of surgery and reducing the risk of recurrence (11). In addition, small GIST is usually treated by clinicians as a general benign tumor. However, once its MI > 5 or even 10/HF, it may also be highly invasive and dangerous; thus, it is obviously not suitable to apply the watch-and-wait treatment strategy. Nevertheless, the accurate prediction of tumor MI is of great significance to evaluate the risk of tumor recurrence and guide the treatment strategy before and after surgery.

Morphological information about tumors can be obtained through endoscopic ultrasonography, computerized tomography (CT), and magnetic resonance imaging (MR); hence, they can be used as a basis to determine the location and size of GIST and indicate the occurrence of rupture or hemorrhage before surgery (12–14). Some prior recent CT-based studies have correlated the morphological features of GIST with the NIH risk classification, prediction of mutation status, and prognosis (15, 16). In clinical practice, CT may be the favored imaging method for GIST preoperative assessment, but MR may provide more tumor information because of its multi-sequence advantage. However, whether CT or MR, the advancements in these conventional imaging methods are limited by subjective human eye observation, which does not provide enough information on the internal heterogeneity of tumors. Moreover, it is difficult to characterize the MI of tumors, which represents important pathological information.

Radiomics was first proposed by Lambin in 2012. It emphasizes the high-throughput extraction of image information (including shape, gray scale, and texture) from medical images and adopts traditional statistical models such as support vector machine, random forest, and XGBoost to achieve tumor segmentation, feature extraction, and model establishment (17). Using radiomics, researchers can transform image information into a large number of features for a quantitative study, which has been widely used in tumor grading, staging, and prognosis research (18–20). The concept of deep learning (DL) was proposed by Hinton et al. in 2006, which is a new field in machine learning research. Its motivation lies in the establishment of neural networks that simulate the analysis and learning process of the human brain, so as to interpret image data by imitating the mechanism of the human brain (21). Unlike radiomics, which relies on predefined artificial features, deep learning algorithms can extract more abstract high-dimensional features in a more automatic way that is not susceptible to subjective influence (22). Therefore, such algorithms have been widely used in the automatic recognition, segmentation, and classification of lung cancer, breast cancer, rectal cancer, and other tumors (23–25). In this study, we trained a convolutional neural network (CNN) classifier based on an integration of two-dimensional (2D) multimodal MR images and three-dimensional (3D) shape-based radiomics features to perform preoperative prediction of mitotic index in GIST.

## Materials and methods

This is a retrospective study, and the patients’ information was anonymized. The ethics committee of our hospital approved the study and waived the need for informed consent from the patients.

### Data

**Figure 1:**
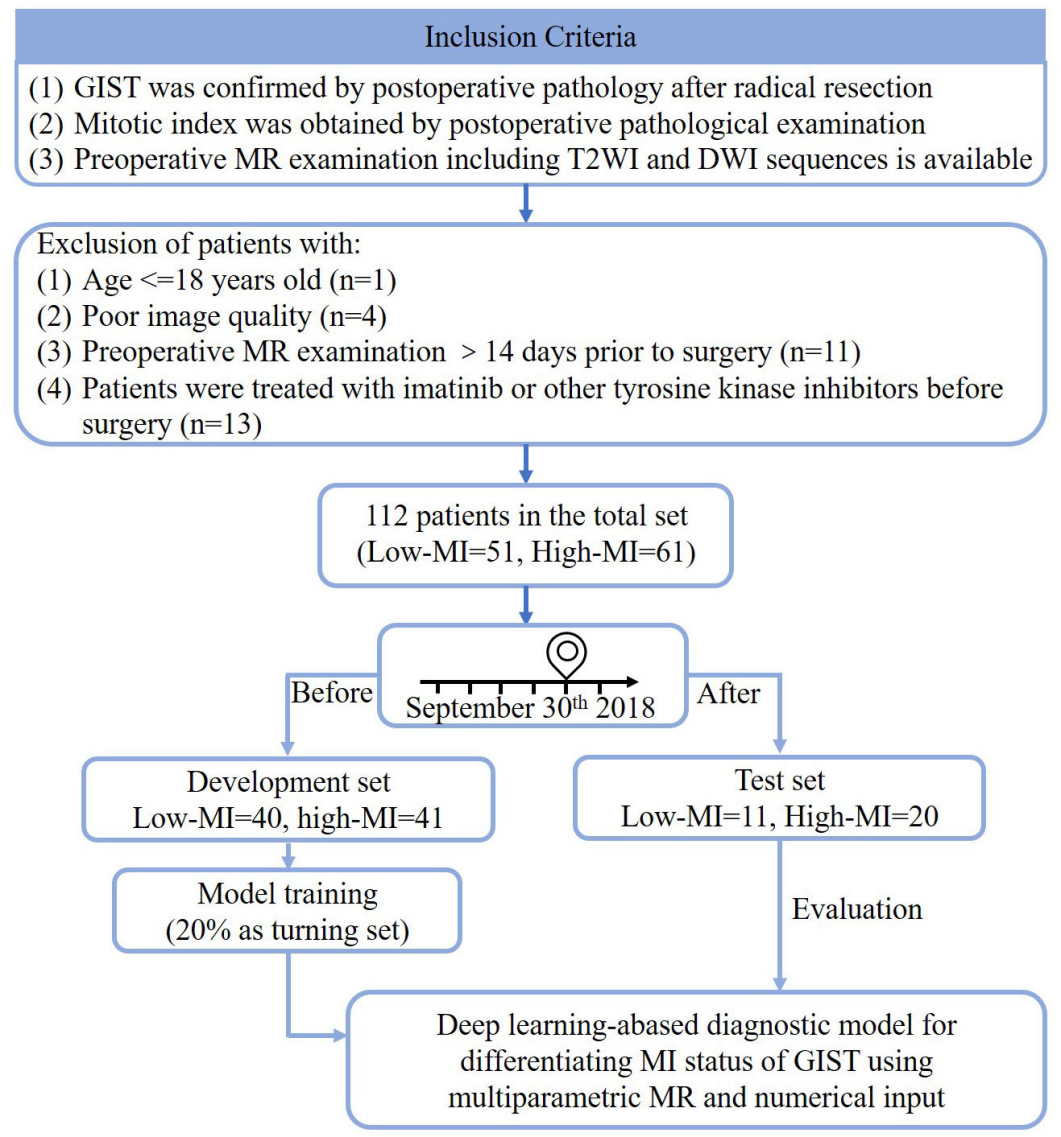
The flowchart of dataset setup. Low-MI, low mitotic index; High-MI, high mitotic index.

A total of 141 patients who were newly diagnosed with GIST and underwent MR examination in our hospital from January 2013 to May 2022 were initially enrolled. The inclusion criteria were as follows: 1) GIST was confirmed by postoperative pathology after radical resection in our hospital; 2) mitotic index was obtained through postoperative pathological examination; 3) preoperative MR examination is available, including T2-weighted imaging (T2WI) and diffusion-weighted imaging (DWI) sequences. The exclusion criteria were as follows: 1) preoperative MR examination occurred more than 14 days before surgery; 2) two radiologists with 5 years of experience in the diagnosis of abdominal and pelvic MR evaluated the image quality and excluded those whose image quality was too poor to delineate the region of interest due to motion or other artifacts; 3) the patients were treated with imatinib or other tyrosine kinase inhibitors before surgery; 4) the patients were younger than 18 years. The patient inclusion process is shown in **Figure 1**. Then, based on postoperative pathology results and modified NIH risk classification criteria (6), the patients were classified into the group with the low mitotic index (MI ≤ 5/50 HPFs, 55 patients) and the group with high mitotic index (MI > 5/50 HPFs, 68 patients). The 2008 modified NIH risk classification criteria are discussed in detail in **Supplementary Table 1**. The data were divided into the development set, consisting of 81 patients who were diagnosed between January 2013 and September 2018, and the test set, consisting of 31 patients who were diagnosed between October 2019 and May 2022.

### Image acquisition and processing

All images were scanned using a 1.5T Siemens Avanto MR system (Siemens, Munich, Germany) equipped with an eight-channel phased-front coil dedicated to the abdomen. In order to reduce gastrointestinal motion artifacts, the patients were instructed to abstain from water and food for 4 h before the scan. The imaging sequences included coronal fast imaging, employing the steady-state acquisition (FIESTA) sequence, axial fat-suppression T2WI, axial DWI, and axial in-phase and out-of-phase T1-weighted imaging (T1WI). DWI was collected by echo-planar imaging (EPI), with b values of 0 and 800. The respiratory trigger technique was used for T2WI and DWI, and the end-expiratory breath-holding method was used for FIESTA and T1WI scans to reduce respiratory motion artifacts. **Table 1** lists the detailed image acquisition parameters.

**Table 1:** MRI protocols.

Image acquisition parameter	Parameter values			
	FIESTA	T2WI	DWI	T1WI
Acquisition plane	Coronal	Axial	Axial	Axial
**Fat saturation**	No	Yes	Yes	No
**TR/TE (ms)**	3.63/1.82	2,000/96	4,600/63	75/2.38,4.79
**Angle (°)**	60	70	150	70
**Slice thickness (mm)**	5	6	6	6
**FOV (mm^2^)**	350 × 350	379 × 284	379 × 308	380 × 320
**Matrix**	512 × 460	384 × 202	192 × 128	320 × 189
**Voxel size (mm^3^)**	1.0 × 1.0 × 5.0	1.0 × 1.0 × 6.0	2.0 × 2.0 × 6.0	1.2 × 1.2 × 6.0
**Interslice gap**	10%	10%	10%	10%
**Delay (s)**				
**Scan time (s)**	12	165	97	69
**b-Value (s/mm^2^)**			0, 800	

FIESTA, fast imaging employing steady-state acquisition; T2WI, T2-weighted imaging; DWI, diffusion-weighted imaging; T1WI, T1-weighted imaging; TR, repetition time; TE, echo time.

### Region of interest segmentation and three-dimensional shape feature extraction

The images of all patients were downloaded in the digital imaging and communications in medicine (DICOM) format from the picture archiving and communication system (PACS) of our hospital. Apparent diffusion coefficient (ADC) maps were registered to T2WI images using the Statistical Parametric Mapping software v.12 (SPM12, University College London). A radiologist with more than 5 years of experience in abdominal and pelvic MR diagnoses segmented the entire tumor in three dimensions on T2WI images, such that the segmentation was strictly along the edges of the tumor and included areas of necrosis and cystic degeneration. In addition, the maximum diameter of the tumor was measured, and the tumor location was recorded during segmentation. The abovementioned information was confirmed and corrected by another radiologist with 10 years of experience in abdominal and pelvic MR imaging. In case of any disagreement, consultation continued until an agreement was reached.

Shape radiomics features were extracted using the PyRadiomics package (https://www.radiomics.io/pyradiomics.html), which contained 14 features, as follows: mesh volume, voxel volume, surface area, surface area to volume ratio, sphericity, maximum 3D diameter, maximum 2D diameter (slice), maximum 2D diameter (column), maximum 2D diameter (row) major axis length, minor axis length, least axis length, elongation, and flatness. The definitions and calculation methods of each of these features can be found on the package documentation page https://pyradiomics.readthedocs.io/en/latest/features.html#module-radiomics.shape.

### Convolutional neural network classifier for mitotic index status prediction

**Figure 2:**
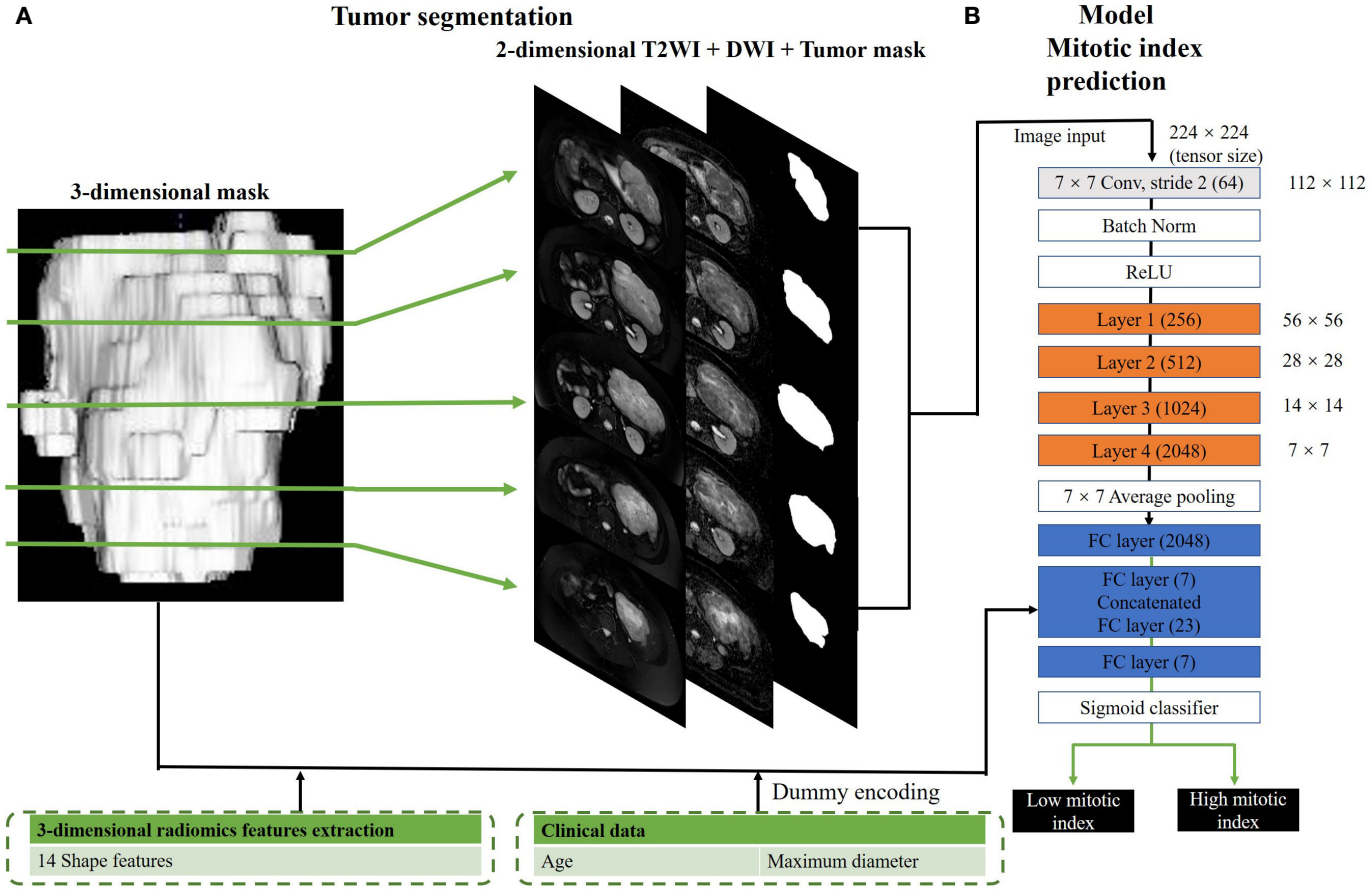
Hybrid model for mitotic index prediction. **(A)** shows the process of 3-dimensional and 2-dimensional image segmentation. We convert a three-dimensional mask to several two-dimensional masks. **(B)** shows the structure of hybrid mitotic index prediction model. In this model, layers 1–4 consisted of three, four, six, and three residual blocks, with each block containing 3 × 3 convolution once and 1 × 1 convolution twice.


The CNN structure is shown in **Figure 2**. The CNN classifier used in this study is derived from the famous 50-layer ResNet structure (hereinafter referred to as conventional ResNet). As shown in **Supplementary Figure 1**, the network structure contained the initial 7 × 7 convolution and layers 1 to 4 comprising three, four, six, and three residual blocks, such that each residual block had one 3 × 3 convolution and two 1 × 1 convolutions. For the hybrid model, we included an additional fully connected layer to the conventional ResNet, which used additional image input and numerical input. The image input to the hybrid model comprised axial T2WI and ADC and tumor masks with the size of 128 × 128. To train a model with a high performance given the insufficient sample size, we selected all the images containing GIST for each patient, instead of a certain layer of images. Therefore, based on tumor segmentation, there may be multiple axial sections per patient, which would be used for the development and testing of classification models. As for the numerical input to the hybrid model, it included 14 morphologic features based on general imaging as well as the patient’s age and tumor diameter. Before adding the above features to the neural network, we standardized them according to the following formula:

$$\vec{{\text{x}}_{n}}=\frac{\vec{{\text{x}}_{n}}-\bar{{\text{x}}_{n}}}{\sqrt{x_{1n}^{2}+x_{2n}^{2}+\ldots +x_{mn}^{2}}}$$

where $\vec{{\text{x}}_{n}}$ is the *n*th feature and m is the number of samples.

The training process of the CNN classifier is discussed in detail below. First, the DICOM image was converted to PNG format, which was used for the training and validation of the CNN model. Since our input data size is 384 × 202, which is bigger than the original residual neural network (224 × 224), the image and mask were resampled. Based on tumor segmentation, all layers of each patient’s tumor were selected as independent samples; this approach might have a better effect on data enhancement than image flipping or rotation. In this way, our convolutional residual neural network and our CNN classifier obtained 891 development samples and 531 test samples. To train our model, the transfer learning method was used, which is widely used in computer vision, for efficient training and accurate classification performance. A weight file obtained by training an ResNet50 network was used on the large ImageNet dataset to extract the features of target datasets, and the model parameters were fine-tuned *via* the target datasets (891 development samples and 531 test samples) to obtain an optimal conventional ResNet model. Then, the weight value of the optimal pretrained conventional ResNet from the initial 7 × 7 convolutional layers to the third layer, and the mixed model was imported and set as untrainable. During the training of the hybrid model, only the weights from the fully connected layers that received shape, age, and maximum diameter as numerical inputs from layer 4 and below were trained to maximize the synergy between the image features from the pretrained weights and numeric inputs. The input images were dynamically enhanced by translation, scaling, rotation, shearing, Gaussian noise, and blur. The Adam optimizer was used to optimize the network (beta1 = 0.9, beta2 = 0.999, initial learning rate = 1e−04), batch size was set to 30, and the maximum training epoch was set to 100, and training was stopped when the lost value of the validation set dropped to a stable level. The resulting model had the lowest validation set loss value. Our CNN model was implemented in PyTorch 1.1.0 (https://pytorch.org) and trained on an NVIDIA Tesla 3080 12 G with a memory of 64 G.

### Cross-validation

To generalize the reliability of the networks, threefold cross-validation was performed on the 111 subjects by randomly shuffling the dataset and distributing it into three groups by stratified randomization (27 subjects for each group: 17 low MI and 20 high MI in Group 1, 17 low MI and 20 high MI in Group 2, and 17 low MI and 21 high MI in Group 3). During each fold of the cross-validation procedure, two of the three groups of subjects were combined as the internal training set, and the remaining group was used as the internal validation set. The internal validation set helped improve network performance during training. Note that each fold of the cross-validation procedure represents a new training phase on a unique combination of the three groups. Network performance was reported on the internal validation set for each fold.

### Statistical analysis

In this study, the predictive performance of the model was studied at the image level and patient level separately such that the results of the image level prediction can finally be used for patient-level prediction. For a certain patient, the average prediction probability of all images was calculated as the prediction probability of the patient. The probability threshold of the calculation accuracy was set as 0.5, so a prediction probability ≥0.5 was classified as high MI, while a prediction probability <0.5 was classified as low MI. The model discrimination ability was evaluated by drawing the area under the receiver operating characteristic curve (AUROC) and the area under the precision–recall curve (AUPRC). In addition to the hybrid and conventional ResNet models, a traditional shape radiomics feature-based classifier was established in this study; the random forest (RF) algorithm was used in the development set, and the 10-fold cross-validation was performed to evaluate the model, with each fold repeated three times using X&Y software (X&Y Solutions, Inc., Boston, MA, USA) based on the R language. The RF algorithm selected and ranked the parameters according to their importance. The constructed “forest” represents the integration of decision trees (DTs) and was trained with the “bagging” method. Bagging methods involve randomly selecting samples of the derivation dataset with replacement, building classifiers, and finally combining the learned models to increase overall performance. In this study, the number of trees in the RF model was 400, with the variables leading to the minimum “out-bagging” error in the model selected as the optimal model. The feature importance was derived from the mean decrease in impurity (MDI). When the RF model has the best effect, the hyperparameters are set as follows: max_depth = 400, max_features = 4, min_sample_leaf = 1, min_sample_split = 2, and *n*_estimators = 400. In order to evaluate whether the hybrid model achieved better diagnostic efficiency, the DeLong test was used to compare AUROC values (26). A p-value <0.05 was considered statistically significant. Statistical analysis was performed using the R software (V3.6.1).

## Results

### Characteristics of the study population

The clinical characteristics of 112 patients are summarized in **Table 2**. The number of patients with low and high MI was 40 and 41 in the development set and 11 and 20 in the test set, respectively. There was no significant difference in the proportion of patients with high MI between the development and test sets (p = 0.186). In the development set, there was a significant difference in age between patients with high and low MI, such that patients with high MI were older (p = 0.032). In the test set, no significant age difference was observed (p = 0.438). In both the development set and test set, there was no significant difference between the high MI group and low MI group in terms of sex (p = 0.224 and p = 0.709, respectively), but the tumor diameter was significantly larger in the high MI group (p < 0.001 and p = 0.003, respectively).

**Table 2:** Patient characteristics.

	Development set (*n =* 81)			Test set (*n =* 31)		
	Low MI (*n =* 40)	High MI (*n =* 41)	p	Low MI (*n =* 11)	High MI (*n =* 20)	p
**Age (years)**			**0.032**			0.438
**Mean ± SD**	52.9 ± 12.8	60.7 ± 18.8		53.7 ± 16.0	59.5 ± 21.0	
**Sex**			0.224			>0.999
**Male**	19 (47.5%)	25 (61.0%)		5 (45.5%)	10 (50.0%)	
**Female**	21 (52.5%)	16 (39.0%)		6 (54.5%)	10 (50.0%)	
**Tumor site**			0.320			>0.999
**Gastric**	21 (52.5%)	17 (41.5%)		4 (36.4%)	8 (40.0%)	
**Non-gastric**	19 (47.5%)	24 (58.5%)		7 (63.6%)	12 (60.0%)	
**Diameter (cm)**			**<0.001**			**0.003**
**Mean ± SD**	6.1 ± 1.9	10.8 ± 3.6		4.3 ± 2.5	9.6 ± 6.3	

Data are presented as mean ± SD or number (percentage). Independent samples t-test was applied in continuous variables. Chi-squared test or Fisher’s exact test was applied to categorical variables. Bold type indicates statistically significant difference.

Low MI, low mitotic index; High MR, high mitotic index.

### Model evaluation

**Figure 3:**
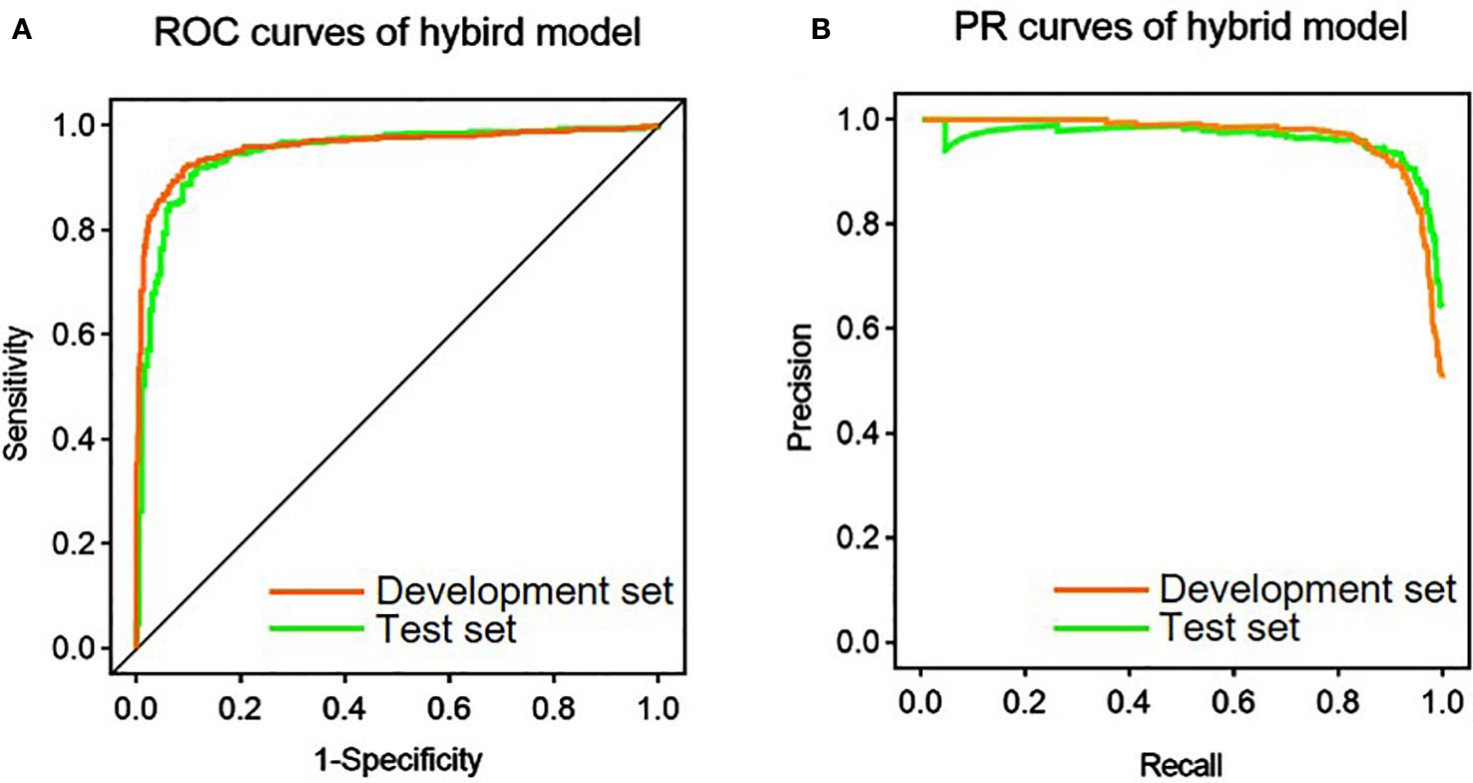
Performance of the hybrid model in the prediction of mitotic index. **(A)** Receiver operating characteristic (ROC) curves of the hybrid model in the development and test set. **(B)** Precision-recall (PR) curves of the hybrid model in the development and test set.

After the image was provided as an input, the conventional ResNet was pretrained for 30 epochs. Among the 14 shape features, the following four features were screened out by the RF algorithm: Elongation, Maximum 2D Diameter row, Sphericity, and Surface Volume Ratio. The variable importance of the shape features and their different distributions according to MI are shown in **Supplementary Figures 2 and 3**, respectively. The abovementioned four features along with age and maximum tumor diameter were used as the numerical input to the hybrid model. Then, part of the weights was imported from the pretrained conventional ResNet and fine-tuned by 30 epochs to produce the hybrid model. **Table 3** and **Figure 3** show the performance of the hybrid model in the development set and test set. At the image level, the AUROC, AUPRC, and accuracy were 0.960, 0.968, and 91.4%, respectively, in the development set and 0.947, 0.964, and 90.8, respectively, in the test set. In addition, with the average probabilities from multiple samples per patient, the hybrid model also showed good discrimination ability at the patient level. It achieved AUROC, AUPRC, and accuracy of 0.913, 0.887, and 91.4%, respectively, in the development set and 0.930, 0.941, and 93.6%, respectively, in the test set.

**Table 3:** Diagnostic performance of the hybrid model for the prediction of mitotic index.

	Development set		Test set	
	Per slice	Per patient^a^	Per slice	Per patient^a^
**AUROC (95% CI)**	0.960 (0.947–0.973)	0.913 (0.851–0.975)	0.947 (0.927–0.968)	0.930 (0.828–1.000)
**AUPRC (95% CI)**	0.968 (0.956–0.977)	0.887 (0.787–0.954)	0.964 (0.930–0.978)	0.941 (0.792–1.000)
**Acc (95% CI)**	91.4 (89.3–93.0)	91.4 (83.2–95.8)	90.8 (88.0–93.0)	93.6 (79.3–98.2)
**Sen (95% CI)**	91.6 (88.5–93.9)	92.7 (79.0–98.1)	92.1 (88.5–94.6)	95.0 (73.1–99.7)
**Spe (95% CI)**	91.1 (88.0–93.5)	90.0 (75.4–96.7)	88.5 (82.9–92.5)	90.9 (57.1–99.5)
**PPV (95% CI)**	91.4 (88.3–93.7)	90.5 (76.5–96.9)	93.4 (90.1–95.7)	95.0 (73.1–99.7)
**NPV (95% CI)**	91.3 (88.2–93.7)	92.3 (78.0–98.0)	86.2 (80.4–90.6)	90.9 (57.1–99.5)

AUROC, area under the receiver operating characteristics curve; AUPRC, area under the precision–recall curve; Acc, accuracy; Sen, sensitivity; Spe, specificity; PPV, positive predictive value; NPV, negative predictive value.

a Since each patient yielded multiple tumor slices, the diagnostic accuracy per patient was calculated from the mean value of the all-predicted probabilities per patient.

The performance evaluation results of conventional ResNet, shape radiomics classifier, and prediction using age plus diameter are shown in **Table 4**. In the development set, the conventional ResNet (per slice), conventional ResNet (per patient), shape radiomics classifier, and age plus diameter achieved AUROCs of 0.951, 0.889, 0.677, and 0.698, respectively; AUPRCs of 0.960, 0.871, 0.665, and 0.761, respectively; and accuracies of 0.899, 0.889, 0.680, and 0.716, respectively. In the test set, they achieved AUROCs of 0.927, 0.880, 0.754, and 0.659, respectively; AUPRCs of 0.929, 0.918, 0.851, and 0.824, respectively; and accuracies of 0.887, 0.871, and 0.772, respectively.

**Table 4:** Diagnostic performance of the conventional ResNet, shape radiomics classifier, and age plus diameter in the prediction of mitotic index.

	Dataset	AUROC (95% CI)	AUPRC (95% CI)	Acc (95% CI)
**Conventional ResNet (per slice)**	Development set	0.951 (0.937–0.966)	0.960 (0.947–0.970)	89.9 (87.8–91.7)
	Test set	0.927 (0.901–0.953)	0.929 (0.880–0.959)	88.7 (85.7–91.1)
**Conventional ResNet** (per patient)^a^	Development set	0.889 (0.820–0.958)	0.871 (0.769–0.946)	88.9 (80.2–94.0)
	Test set	0.880 (0.760–1.000)	0.918 (0.746–0.979)	87.1 (71.2–94.9)
**Shape radiomics classifier**	Development set	0.677 (0.641–0.712)	0.665 (0.629–0.709)	68.0 (64.9–71.0)
	Test set	0.754 (0.712–0.797)	0.851 (0.823–0.876)	77.2 (73.5–80.6)
**Age plus diameter**	Development set	0.698 (0.574–0.803)	0.761 (0.657–0.852)	71.6 (61.0–80.3)
	Test set	0.659 (0.465–0.853)	0.824 (0.690–0.915)	61.3 (43.8–76.3)

AUROC, area under the receiver operating characteristics curve; AUPRC, area under the precision–recall curve.

a Since each patient yielded multiple tumor slices, the diagnostic accuracy per patient was calculated from the mean value of the all-predicted probabilities per patient.

### Model explanation

#### Comparison of the hybrid model with other models

The comparison results of AUROCs and accuracies between the hybrid model and age plus diameter, shape radiomics classifier, and conventional ResNet are shown in **Supplementary Table 2** and **Supplementary Figure 4**. In both the development set and the test set, the hybrid model outperformed the prediction of age plus diameter, and there were significant differences in AUROC and accuracy between the two models (all p < 0.05 for accuracy and AUROC). In addition, the hybrid model was also superior to the shape radiomics classifier (p < 0.05 for both accuracy and AUROC). However, compared with conventional ResNet, the hybrid model has slightly higher AUROC and accuracy, but the difference between them is not significant.

#### Ablation analysis

The results of the ablation analysis are discussed in detail in **Supplementary Table 3**. Compared with the conventional ResNet, as we reduced the number of input images per patient and reduced the sequences or masked tumor area, we observed a decrease in the diagnostic performance, with accuracies, AUROCs, and AUPRCs at 70.4%–84%, 0.840–0.704, and 0.676–0.814, respectively, in the development set and 61.3%–83.9%, 0.639–0.834, and 0.746–0.873, respectively, in the test set. When we masked the tumor area from image inputs, the lowest diagnostic performance was achieved, with accuracy, AUROC, and AUPRC of 61.7%, 0.618, and 0.602, respectively, in the development set and 54.8%, 0.548, and 0.676, respectively, in the test set.

### Cross-validation

To ensure that the performance of the hybrid model was not due to the random selection of the internal test set, we performed a patient-level threefold cross-validation on the entire cohort (*n* = 112). In the internal validation set, the mean AUROC was 0.910 (range, 0.896–0.927) and 0.903 (range, 0.849–0.980) in the hybrid model (per slice) and hybrid model (per patient), respectively (**Supplementary Table 4**), similar to those in the test set. The cross-validation results show that the hybrid model has good robustness.

## Discussion

In this study, based on a ResNet50 CNN, we developed a hybrid model to predict the MI status of GIST patients. The CNN integrating 2D tumor signal intensity, 3D tumor shape, patient age, and tumor size showed good predictive power in both the development and test sets.

GIST mitotic index is an important indicator of metastasis and prognosis, which is independent of the tumor size and location; this led to the integration of this indicator in the NIH system. Although radical resection is still the most commonly used standard treatment for GIST, due to the high risk of postoperative recurrence for patients with high MI, surgical resection following neoadjuvant therapy may improve the prognosis (27). Preoperative prediction of MI potentially helps in setting the treatment plan, which leads to the investigation of radiological findings to predict the MI status. A previous CT study showed that GIST with high MI and high-risk grade is more prone to internal necrosis, neovascularization, and peripheral invasion, while low MI tumors have more regular morphology and clearer boundaries with the surrounding tissues (28). In addition, an MR study with higher soft tissue resolution showed that tumor enhancement was significantly stronger in patients with high MI compared with patients with low MI, which may be related to the formation of new tumor vessels inside (29). Some studies tried to evaluate the grading of GIST using a DWI-based ADC map and PET-CT parameter map, and they found the ADC value to be negatively correlated with the grading of the GIST tumor, while the metabolic rate was negatively correlated with it (30, 31). Changes in ADC caused by targeted therapy may be related to a variety of cell death mechanisms, including mitotic catastrophe, which indicates that ADC can provide more information to evaluate mitosis from a therapeutic perspective (32). Therefore, the ADC map was taken as one of the sequences of the multimodal study in this study.

Radiomics can be used to obtain high-level features of tumor images, which can reflect the heterogeneity of tumors and provide a basis to evaluate biological behavior (33). A recent enhanced CT-based study found a close relationship between the mitotic number and 14 radiomics features of GIST, which suggests that it may be another possible method to predict the number of GIST (34). However, this study was based only on 2D images of the maximum cross-section of the tumor, which did not fully obtain the overall information about the tumor. Moreover, the study only included enhanced CT images, with a single type of image. As a result, the accuracy of its prediction model in the test set was only 85.4%. In this study, the accuracy of the hybrid model reached 93.6% after including the information at all layers of the tumor.

Deep learning refers to a technology that combines low-level features to form more abstract high-level features or categories and then learns effective features from a large number of input elements and uses these features to perform classification, regression, and information retrieval. There are many kinds of DL models, among which CNN is most widely used in the field of medical imaging. Unlike traditional radiomics based on manual feature extraction, high-throughput image features can be directly extracted from deep neural networks (DNNs) without additional feature extraction operations; thus, no additional error occurs in the analysis due to feature calculation, and the effectiveness of the feature is only related to the segmentation quality (35, 36). At present, CNN has been successfully applied in many aspects, such as genotype prediction, preoperative staging, lymph node metastasis prediction, and prognosis evaluation of malignant tumors (37–39). The application of the DL algorithm to extract image information can overcome the influence of observer subjectivity.

Researchers have begun to explore the application of DL in the diagnosis and evaluation of GIST. Previously, a DL model for predicting the mitotic index of GIST was preliminarily established by providing venous images as input into CNN. The results showed that the image-based DL model could evaluate the MI of GIST before surgery (40). However, the generalization ability of the model proposed in their study was not high, and the area under the curve (AUC) in the internal test set was only 0.771–0.800. In our study, the AUC of the conventional ResNet model reached 0.880–0.927 in the test set, while the hybrid model achieved an even better predictive ability, with an AUC of 0.930–0.947. The reason may be that MR has a higher soft tissue resolution as compared with CT, so images may contain more information, and the extracted DL features may have better discrimination ability. The input images in this study were multimodal MR images (including T2WI and DWI images). Previous studies have confirmed that multimodal images can improve the final effect of the DL model. In a previous study, researchers also fed endoscopic ultrasound images into neural networks for auxiliary diagnosis of GIST and gastrointestinal leiomyoma. Their study showed that the two tumor types could not be distinguished based on naked-eye observation, and the accuracy was only 63%, while the accuracy of the CNN system reached 86.98% (41). Another study confirmed that an EUS–CNN system can be helpful not only for less-experienced endoscopists but also for experienced ones (42).

ResNet50 CNN was selected as the basic model in this study. ResNet50 is a network framework of residual learning that solves the degradation problem of decreasing accuracy caused by increasing the network depth. Compared with previous models, the residual network is easier to optimize and can derive accuracy from a significantly increased depth (43). Many previous studies have used this network to classify tumors and achieved good results (44–46). The transfer learning method was adopted, and a fully connected layer was added to the hybrid model. The results of multi-slice CT images can better reflect the overall biological behavior and mitotic rate of the tumor than that of single-slice CT images (47).

In addition to the multi-modal image input, the construction process of the proposed hybrid model proposed was different from that of the image-based CNN model reported in previous studies (40). While the hybrid model combined shape features and clinical indicators, in order to ensure the robustness of the model, only shape features in traditional radiomics were selected to establish the model. The main factor limiting the repeatability of radiomics features is that the extraction results of first-order and texture features depend on the range and number of bins of signal intensity, and there is currently no accepted standard to set the signal strength-related parameters (48, 49). Unlike the intensity feature, the morphological feature is independent of the abovementioned settings and can thus remain stable across studies. This improves the stability of the research model.

The hybrid model fuses the 3D tumor morphology and mitotic-related clinical indicators (age and tumor size) with the CNN model, thus producing an enhanced model performance compared with image-based CNN alone. Previous studies showed that age and tumor size are independent risk factors for prognosis in GIST patients (50). In this study, there were significant differences in the age and maximum diameter between the high MI group and low MI group. It was previously shown that older patients with meningiomas are more likely to have more active mitosis and larger tumors, which indicates that they have faster tumor division and may have a higher MI (51). However, the relationship between MI and the factors of age and tumor size needs to be further confirmed in GIST. Despite the differences between groups, the prediction efficiency of these two indicators alone for MI is very low, which also indicates that it is difficult to use only clinical indicators for the MI status of tumors in clinical practice, and we need to combine more indicators reflecting the internal heterogeneity of the tumor.

This study used radiomics and deep learning analysis based on MR plain scan images to predict mitosis in GIST. However, due to the limited time resolution, MR is highly susceptible to respiratory movement and intestinal peristalsis during abdominal imaging, which limits its application in GIST assessment. Compared with MR, CT is more widely used in the preoperative evaluation of GIST in clinical at present, which has the advantages of low cost, short examination time, and low susceptibility to motion artifacts (52). However, plain CT has the inherent defect of insufficient soft tissue resolution, so contrast-enhanced CT is often adopted for preoperative evaluation of GIST, which may increase the renal burden and allergy risk of patients. In addition, CT imaging is single-parameter imaging based on tissue density, which provides limited information. However, MRI has the advantage of multi-sequence and arbitrary angle imaging, which is more conducive to displaying the relationship between tumors and surrounding organs from different angles. Given the above advantages of MR, GIST can be accurately assessed clinically using only MR plain scan sequences (53). In addition to higher tissue contrast, the application of functional imaging sequences such as DWI can provide microscopic information about the tumor from the tissue level and even the cell level (30). Radiomics or deep learning features based on such specially weighted images may better reflect the heterogeneity of the tumor.

This study has some limitations that merit discussion. First, the sample size of this study is small, so future studies should continue the data collection and use a larger sample size. Second, this study is a single-center study. Although internal verification has been performed, the repeatability and generalization ability of the model should be further verified by external datasets. Finally, because GIST is irregular in shape and may occur in any part of the digestive tract and its adjacent tissues and organs are complex, it is difficult to achieve automatic segmentation of the tumors. In this study, manual segmentation was adopted, which is more difficult but more accurate.

In conclusion, we developed a deep learning-based model that used radiomics and clinical features to reliably predict the MI status in GIST based on conventional, unenhanced MR images. Our model is expected to serve as a practical tool for the non-invasive characterization of GIST to support personalized treatment plans.

## Data availability statement

The raw data supporting the conclusions of this article will be made available by the authors, without undue reservation.

## Ethics statement

The studies involving human participants were reviewed and approved by The Ethics Committee of the First Hospital of Qinhuangdao. Written informed consent for participation was not required for this study in accordance with the national legislation and the institutional requirements. Written informed consent was not obtained from the individual(s) for the publication of any potentially identifiable images or data included in this article.

## Author contributions

DL, ZW, LL, and YC designed and coordinated the study. LY, DD, TZ, HY, JD, and YF carried out the experiment and data processing and drafted the manuscript. All authors gave the final approval for the publication.

## Funding

The research leading to these results has received funding from the National Science Foundation of China (81871029).

## Conflict of interest

The authors declare that the research was conducted in the absence of any commercial or financial relationships that could be construed as a potential conflict of interest.

## Publisher’s note

All claims expressed in this article are solely those of the authors and do not necessarily represent those of their affiliated organizations, or those of the publisher, the editors and the reviewers. Any product that may be evaluated in this article, or claim that may be made by its manufacturer, is not guaranteed or endorsed by the publisher.
