# Supplementary material for: Deep learning and radiomics to predict the mitotic index of gastrointestinal stromal tumors based on multiparametric MRI
Source: Front Oncol. 2022 Nov 23;12:948557. doi: 10.3389/fonc.2022.948557 (PMC9727176; doi:10.3389/fonc.2022.948557)
Supplement: Supplementary file 1 [file DataSheet_1.docx]

# Supplementary Material

**Deep learning and radiomics to predict the mitotic index of gastrointestinal stromal tumor based on multiparametric MRI**

Linsha Yang, Dan Du, Tao Zheng, Lanxiang Liu, Zhanqiu Wang, Juan Du, Huiling Yi, Yujie Cui, Defeng Liu, Yuan Fang

目录

[Supplementary Material 1](#_Toc115595817)

[eDocument 1. Ablation analysis 3](#_Toc115595818)

[eFigure 1. Architectures of the conventional ResNet 4](#_Toc115595819)

[eFigure 2. Relative variable importance 5](#_Toc115595820)

[eFigure 3. Heatmap of radiomics classifier 6](#_Toc115595821)

[eFigure 4. ROC among different prediction models 7](#_Toc115595822)

[eTable 1. Modification of risk classification guideline for patients with GIST 8](#_Toc115595823)

[eTable 2. Comparison of the performance of the hybrid model with age plus diameter, shape radiomics and Conventional ResNet for the MI prediction 9](#_Toc115595824)

[eTable 3. The ablation analysis results with image inputs in the prediction of *MI*. 10](#_Toc115595825)

[eTable 4. Relationship between cross validation folds and performance of the hybrid model 11](#_Toc115595826)

## eDocument 1. Ablation analysis

For ablation analysis to potentially explain which image sequence and regions are relevant for *mitotic index* prediction, the conventional ResNet was re-trained with the image inputs that were partially ablated as follows: for image slice ablation, only one or three slices out of the five slices per patients that were used for final model training were used, yielding smaller sample size. For image channel ablation, one or two out of three image channels (i.e. T2, ADC and tumor masks) were used for model training. For image part ablation, either tumor area or nontumor brain tissue area was masked from the input images.

## eFigure 1. Architectures of the conventional ResNet

Hybrid model was derived from the conventional ResNet (A), that mainly consisted of two types of residual blocks, Res-Block (B), and 2 (C). Conv, convolution.

**
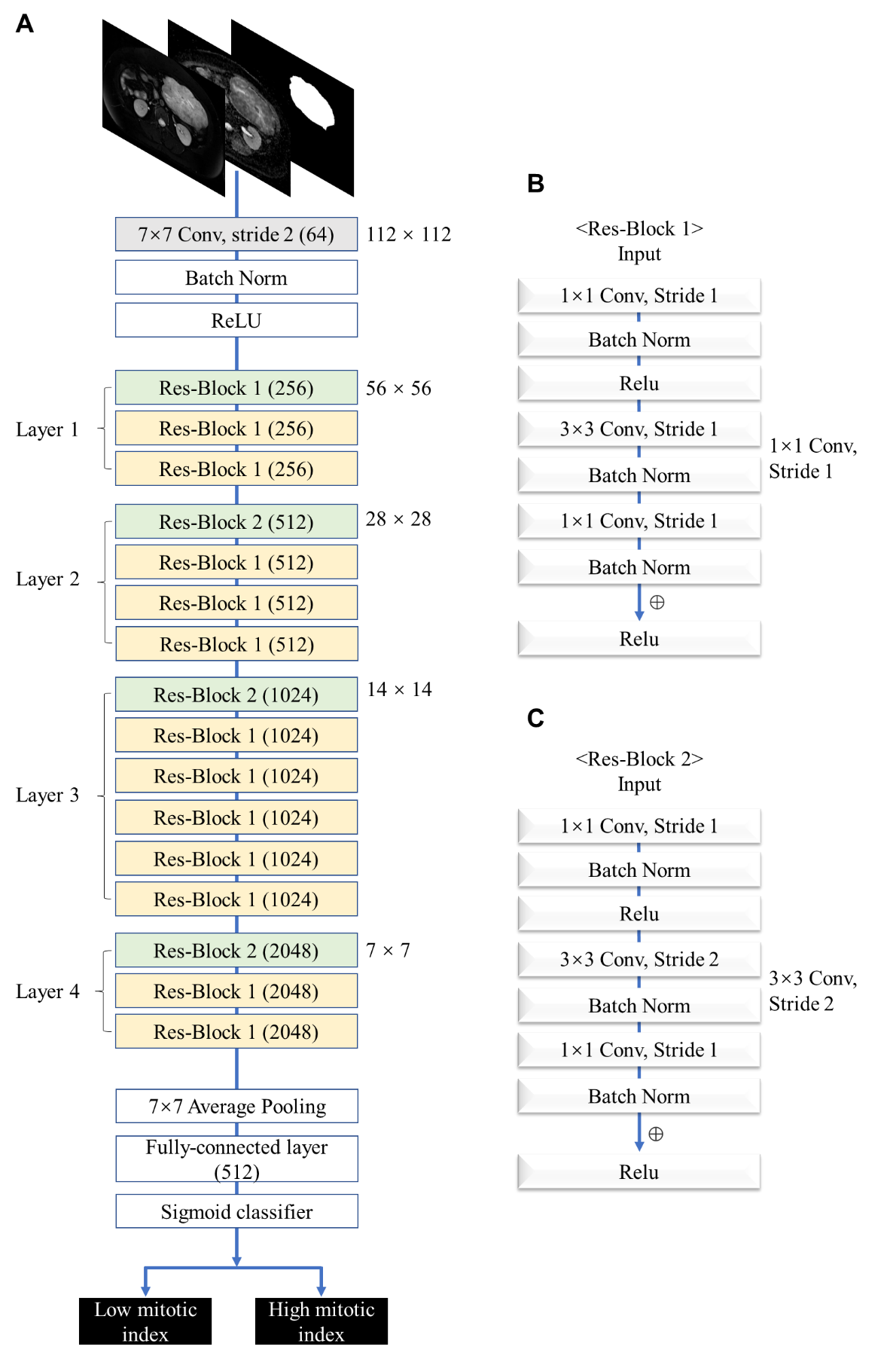
**

## eFigure 2. Relative variable importance

Relative variable importance of the top 4 relevant numeric features in the radiomics classifier in the prediction of MI.

**
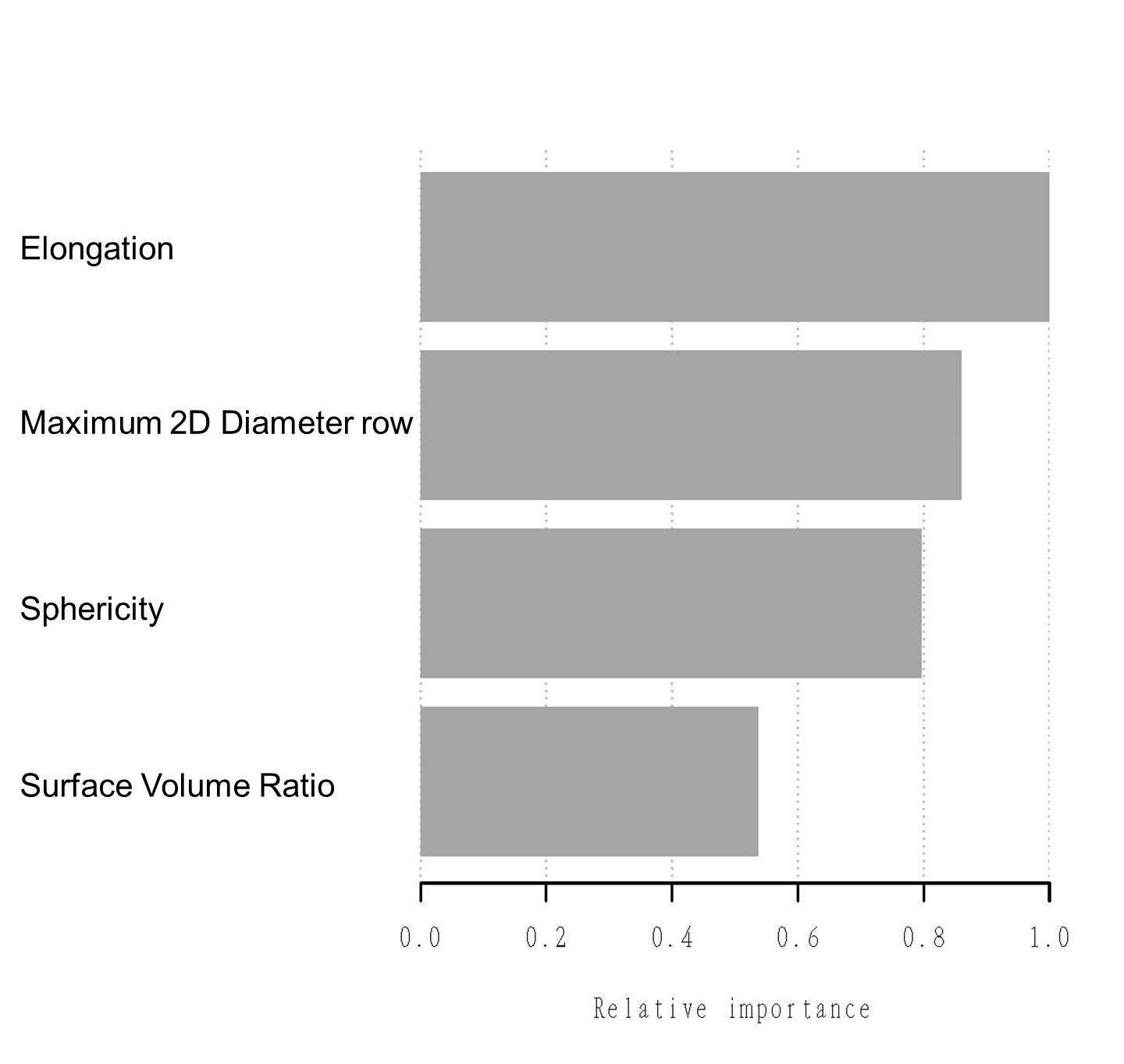
**

## eFigure 3. Heatmap of radiomics classifier

Distribution of the top 4 relevant features of shape radiomics classifier according to the MI status, from the development set (A), and test set (B).


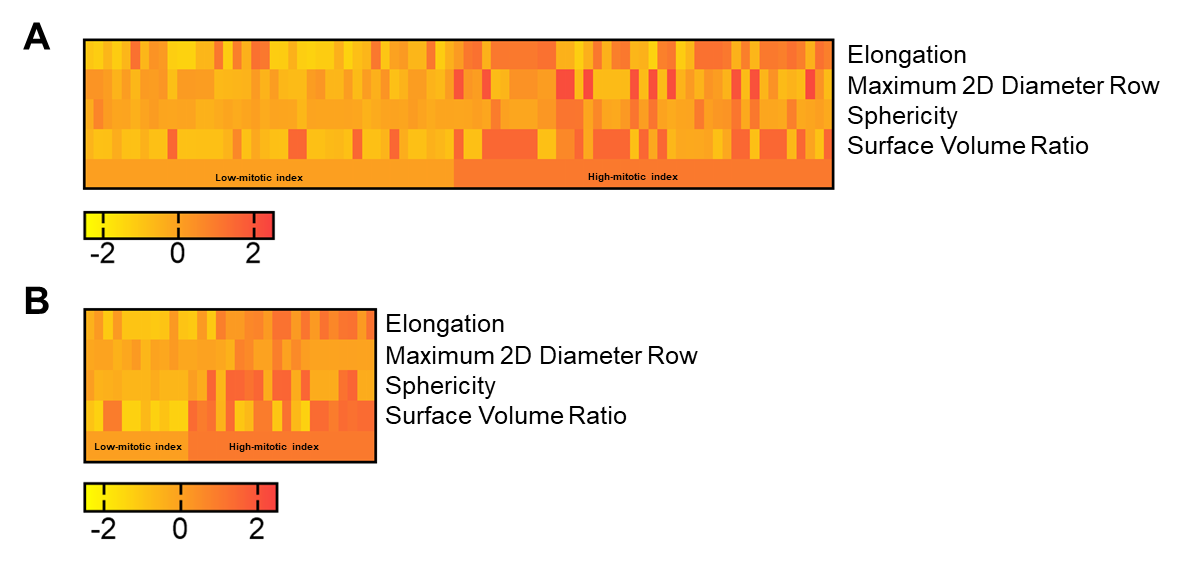


## eFigure 4. ROC among different prediction models

**
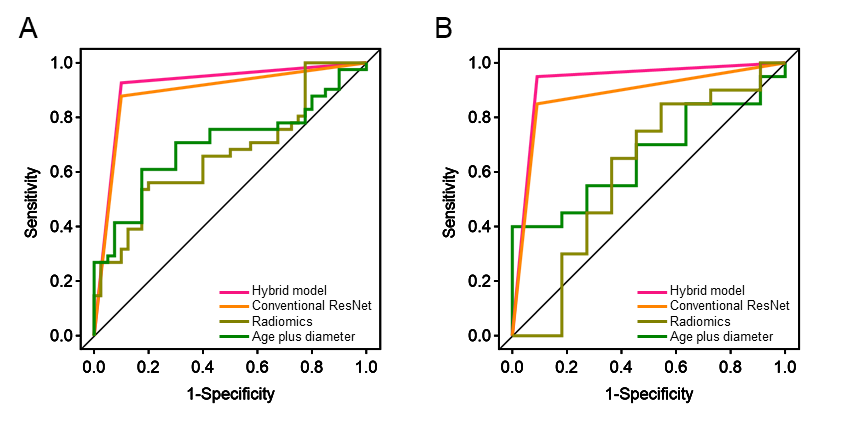
**

Receiver operating characteristic curves among different prediction models in the development (A) and test (B) sets.

## eTable 1. Modification of risk classification guideline for patients with GIST

|  | **Tumor size (cm)** | **Mitotic index (per 50 HPFs)** | **Primary tumor site** |
| --- | --- | --- | --- |
| Very low risk | <2.0 | ≤5 | Any |
| Low risk | 2.1-5.0 | ≤5 | Any |
| Intermediate risk | 2.1-5.0 | >5 | Gastric |
|  | <5.0 | 6-10 | Any |
|  | 5.1-10.0 | ≤5 | Gastric |
| High risk | Any | Any | Tumor rupture |
|  | >10 | Any | Any |
|  | Any | >10.0 | Any |
|  | >5.0 | >5 | Any |
|  | 2.1-5.0 | >5 | Nongastric |
|  | 5.1-10.0 | ≤5 | Nongastric |

## eTable 2. Comparison of the performance of the hybrid model with age plus diameter, shape radiomics and Conventional ResNet for the MI prediction

| **Dataset** | **Model** | **AUROC (95% CI)** | **P value^a^** | **Accuracy (%)** | **P value^b^** |
| --- | --- | --- | --- | --- | --- |
| Development set | Hybrid model | 0.913 (0.851-0.975) |  | 91.4(83.2-95.8) |  |
|  | Age plus diameter | 0.698 (0.574-0.803) | **0.005** | 71.6 (61.0-80.3) | **0.001** |
|  | Shape Radiomics | 0.666 (0.545-0.787) | **<0.001** | 69.1 (58.4-78.1) | **<0.001** |
|  | Conventional ResNet | 0.889 (0.820-0.958) | 0.146 | 88.9 (80.2-94.0) | 0.598 |
| Test set | Hybrid model | 0.930 (0.828-1.000) |  | 93.6 (79.3-98.2) |  |
|  | Age plus diameter | 0.659 (0.465-0.853) | **0.024** | 61.3 (43.8-76.3) | **<0.001** |
|  | Shape Radiomics | 0.605 (0.414-0.775) | **0.019** | 71.0(53.4-83.9) | **0.043** |
|  | Conventional ResNet | 0.880 (0.760-1.000) | 0.152 | 87.1 (71.2-94.9) | 0.671 |

^a^ P value from comparison of accuracies from the hybrid model and age, by an exact binomial test. ^b^ P value from comparison of AUROC values from the hybrid model and age, by the method by DeLong et al. Bold type indicates statistically significant difference.

## eTable 3. The ablation analysis results with image inputs in the prediction of *MI*.

| **Model** | **Development set** | | | **Test set** | | |
| --- | --- | --- | --- | --- | --- | --- |
|  | **Accuracy (%)** | **AUROC (95% CI)** | **AUPRC (95% CI)** | **Accuracy (%)** | **AUROC (95% CI)** | **AUPRC (95% CI)** |
| Reference (original)* | 88.9 (80.2-94.0) | 0.889 (0.820-0.958) | 0.871 (0.769-0.946) | 87.1 (71.2-94.9) | 0.880 (0.760-1.000) | 0.918 (0.746-0.979) |
|  |  |  |  |  |  |  |
| Different No. of axial image samples |  |  |  |  |  |  |
| 3 image samples per patient ^a^ | 84.0 (74.5-90.4) | 0.840 (0.741-0.912) | 0.814 (0.710-0.900) | 83.9 (67.4-92.9) | 0.834 (0.657-0.943) | 0.873(0.731 -0.971) |
| 1 image samples per patient ^b^ | 79.0 (68.9-86.5) | 0.790 (0.686-0.873) | 0.760(0.662-0.854) | 80.7 (63.7-90.8) | 0.809 (0.628-0.927) | 0.860(0.740 -0.968) |
|  |  |  |  |  |  |  |
| Image Sequence |  |  |  |  |  |  |
| T2 + Tumor mask | 77.8 (67.6-85.5) | 0.778 (0.672-0.863) | 0.751 (0.641-0.836) | 71.0 (53.4-83.9) | 0.714 (0.524-0.861) | 0.792(0.662- 0.920) |
| ADC + Tumor mask | 79.0 (68.9-86.5) | 0.790 (0.685-0.873) | 0.753 (0.661-0.847) | 74.2 (56.8-86.3) | 0.739 (0.550-0.879) | 0.806(0.691-0.945) |
| T2 + ADC | 75.3 (64.9-83.4) | 0.753 (0.645-0.842) | 0.719 (0.621-0.807) | 71.0 (53.4-83.9) | 0.734 (0.545-0.876) | 0.819(0.660 -0.933) |
| T2 | 70.4 (59.7-79.2) | 0.704 (0.592-0.800) | 0.671 (0.577-0.769) | 64.5 (47.0-78.9) | 0.664 (0.472-0.822) | 0.762(0.647 -0.911) |
| ADC | 70.4 (59.7-79.2) | 0.704 (0.592-0.800) | 0.676 (0.574-0.778) | 67.7 (50.1-81.4) | 0.709 (0.519-0.857) | 0.804(0.658 -0.920) |
|  |  |  |  |  |  |  |
| Image region |  |  |  |  |  |  |
| Nontumor area masked | 76.5 (66.2-84.4) | 0.766 (0.658-0.853) | 0.735 (0.632-0.827) | 61.3 (43.8-76.3) | 0.639 (0.447-0.802) | 0.746(0.615 -0.880) |
| Tumor area masked | 61.7 (50.8-71.6) | 0.618 (0.503-0.724) | 0.602 (0.511-0.699) | 54.8 (37.8-70.8) | 0.548 (0.360-0.726) | 0.676(0.566-0.821) |

Note: The model performance is described as per patient (not per image sample), based on the mean probability from axial image samples of each patient. *The performance of the conventional ResNet that used the same image inputs of the hybrid model, which comprised all axial image samples per patient, with each image sample consisting of T2, ADC, and tumor mask. ^a^The three image samples per patient were obtained from the axial slice with maximum tumor area, and 2 lower and upper slices from that slice. ^b^The one image samples per patient was obtained from the axial slice with maximum tumor area. AUROC = area under the receiver operating characteristics curve; AUPRC = area under the precision-recall curve.

## eTable 4. Relationship between cross validation folds and performance of the hybrid model

| Model | Fold | AUROC (95% CI) | AUPRC (95% CI) | Accuracy (95% CI) |
| --- | --- | --- | --- | --- |
| Hybrid model (per slice) | Fold 1 | 0.896 (0.870-0.922) | 0.886 (0.837-0.916) | 86.2 (83.2-88.6) |
|  | Fold 2 | 0.907 (0.890-0.924) | 0.909 (0.860-0.958) | 85.5 (83.2-88.4) |
|  | Fold 3 | 0.927 (0.901-0.953) | 0.929 (0.880-0.978) | 93.7 (87.5-100.0) |
|  | MOF | 0.910 (0.887-0.933) | 0.908 (0.859-0.951) | 88.4 (84.6-92.3) |
| Hybrid model (per patient)^a^ | Fold 1 | 0.849 (0.729-0.969) | 0.875 (0.750-1.000) | 84.6 (68.7-100.0) |
|  | Fold 2 | 0.880 (0.760-1.000) | 0.818 (0.781-0.855) | 87.1 (73.2-100.0) |
|  | Fold 3 | 0.980 (0.960-1.000) | 0.828 (0.646-1.000) | 77.1 (61.2-93.0) |
|  | MOF | 0.903(0.816-0.990) | 0.840 (0.726-0.952) | 82.9 (67.7-97.7) |

Note: AUROC, area under the receiver operating characteristics curve; AUPRC, area under the precision-recall curve; MOF, mean of folds

^a^Since each patient yielded multiple tumor slices, the diagnostic accuracy per patient was calculated from the mean value of the all-predicted probabilities per patient.
